# Allergy to Cats: Current Perspectives and Therapeutic Options

**DOI:** 10.1002/clt2.70152

**Published:** 2026-03-03

**Authors:** Pascal Demoly, Myriam Zakariya, Ignacio Dávila, Giuseppe Scibilia, Valeria Ortolani, Javier Domínguez‐Ortega, Karl‐Christian Bergmann, Philippe Gevaert, Alain Didier

**Affiliations:** ^1^ University Hospital of Montpellier and IDESP University Hospital of Montpellier—INSERM—INRIA PreMediCal Montpellier France; ^2^ Clinique de l’Estrée Stains France; ^3^ Allergy Department University Hospital of Salamanca Salamanca Spain; ^4^ Department of Biomedical and Diagnostic Sciences University of Salamanca Salamanca Spain; ^5^ Redes de Investigación Cooperativa Orientadas a Resultados en Salud (RICORS) Red de Enfermedades Inflamatorias (REI) Instituto de Salud Carlos III Madrid Spain; ^6^ Department of Allergy and Clinical Immunology ASST Niguarda Great Metropolitan Hospital Milan Italy; ^7^ Department of Allergy and Clinical Immunology Sacco Hospital Milan Italy; ^8^ Department of Allergy Hospital Universitario La Paz Institute for Health Research (IDIPAZ) Madrid Spain; ^9^ Institute of Allergology Charité‐Universitätsmedizin Berlin Berlin Germany; ^10^ Fraunhofer Institute for Translational Medicine and Pharmacology (ITMP) Immunology and Allergology Berlin Germany; ^11^ Upper Airways Research Laboratory Ghent University Ghent Belgium; ^12^ Department of Pneumoallergology Hôpital Larrey CHU de Toulouse Toulouse France; ^13^ CRISALIS F‐CRIN/INSERM Toulouse France

**Keywords:** allergen immunotherapy, allergic rhinitis, asthma, cat allergy, Fel d 1

## Abstract

Allergic rhinitis (AR) and asthma caused by cat dander have a highly variable prevalence across countries, which can reach 30% of the population in some regions. Cat allergens are widely distributed in the environment, making exposure nearly unavoidable, even in non‐cat‐owning households. Eight cat allergens have been identified, with Fel d 1 and Fel d 4 being particularly associated with the development and severity of asthma. Symptoms can range from mild nasal and eye symptoms to severe asthma exacerbations, with many patients experiencing polysensitization to other allergens. Management usually begins with allergen avoidance and pharmacotherapy, but these approaches are often insufficient. Allergen immunotherapy (AIT), both sublingual (SLIT) and subcutaneous (SCIT), offers a disease‐modifying strategy, though allergen potency, composition, standardization issues, and low prescription rates limit its use. AIT formulations that include allergens beyond Fel d 1, such as Fel d 4, show promise in improving cat‐induced asthma and rhinitis outcomes. Additionally, novel approaches for antigen presentation or combination therapies with monoclonal antibodies may enhance the effectiveness and safety of AIT. To increase treatment success, personalized care using component‐resolved diagnostics to identify sensitization profiles and better education for both physicians and patients are essential in the broader adoption of cat AIT.

**Abbreviations**
AIT: allergen immunotherapy
AR: allergic rhinitis
Cat‐PAD: cat peptide allergy desensitization
CRD: component‐resolved diagnostic
FEV1: forced expiratory volume in one second
HEPA: high‐efficiency particulate air
IR: index of reactivity
RCT: randomized controlled trial
SCIT: subcutaneous immunotherapy
SLIT: sublingual immunotherapy
SPT: skin prick test
TAA: total allergenic activity
TSLP: thymic stromal lymphopoietin
VLP: virus‐like particle

## 1 Introduction

The frequency of allergies has doubled over the last 20 years, and some projections suggest that by 2050 up to half of people may be affected by an allergic disease [1]. Allergic rhinitis (AR) is a respiratory condition that can affect more than a quarter of the population [2, 3]. Its prevalence is geographically diverse, being generally higher in urbanized and developed regions, likely due to heightened allergen exposure, pollution, and lifestyle [3]. The frequency and severity of respiratory allergies have increased over the past 20 years, likely due to urbanization, climate change, and biodiversity loss, and continue to rise [2, 4]. Accordingly, the prevalence and severity of asthma are also globally increasing [5, 6, 7]. Therefore, AR and allergic asthma are major global health concerns associated with considerable individual and societal morbidity, including decreased school performance or loss of work productivity [3, 8].

Intermittent (i.e., seasonal) AR is triggered by pollen and has often been the focus of most epidemiological research; in contrast, the prevalence of persistent (i.e., perennial) AR resulting from indoor allergens such as dust mites and pet dander can likely be underestimated [9]. Among all respiratory allergies, cat dander is the third leading cause of AR after mites and pollens, with a considerable impact on the patient's quality of life [10]. The symptoms of cat‐derived allergy can be ocular, nasal, bronchial, cutaneous, or even systemic. Disease severity can range from relatively mild rhinoconjunctivitis to potentially life‐threatening asthma exacerbations leading to hospitalization. A high proportion (∼30%) of cat‐allergic patients have asthma [11, 12]. Additionally, cat dander sensitization is closely linked to the risk of developing asthma or experiencing more severe asthma [13, 14]. Although controversies remain, it has been recommended that patients with asthma who are sensitized to cat allergens minimize their exposure to reduce the likelihood of an exacerbation [10, 15, 16]. Unlike other allergies (e.g., house dust mites), it is not necessary to be heavily exposed to cat allergens to develop cat allergy symptoms [12]. Several standardized methodologies for the study of cat‐induced AR and allergic asthma have been developed, such as natural exposure cat rooms, allergen exposure chambers, and nasal allergen challenges, in an effort to standardize the clinical evaluation of therapies [17].

While extensive research has been conducted to understand the nature of human allergies to cats, the evaluation and management of clinical symptoms in affected individuals remains challenging. Multifaceted management strategies are typically advised, often based on avoiding or reducing contact with cats and pharmacological approaches to reduce or alleviate symptoms. However, the advice to avoid cats or remove the cat is rarely followed or acceptable for most patients. Despite the high prevalence and burden of the disease, management of cat‐derived respiratory allergy is often suboptimal. The objective of this review is to evaluate the current state of research on cat dander‐derived AR and asthma and to raise awareness of the need for appropriate care for patients allergic to cats.

## 2 Cat Allergy Epidemiology

In many countries, cats are the most common household pets. According to a 2015 online survey of 27,000 people across 22 industrialized countries, 57%, 41%, and 39% of respondents in Russia, France, and the USA, respectively, owned a cat [18]. AR caused by exposure to cat dander is triggered by allergens on microscopic particles from the cat's skin, saliva, or fur shed by cats. These particles are lightweight and sticky, and can easily adhere to clothing, furniture, and air particles, spreading far beyond areas where cats are present [19, 20]. Immunologically significant levels of cat allergens are frequently detected in environments without cats, such as schools, daycare centers, cars, hospitals, churches, cinemas, hotels, as well as on public transportation like trains, buses, and airplanes [20, 21, 22]. Moreover, in many European countries, current legislation permits cats, along with other pets, to travel on public transport, provided certain requirements are met [22]. For these reasons, although cat ownership is a risk factor for cat sensitivity, it is not necessary to have a cat at home to develop cat allergy [23, 24, 25].

Data on the prevalence of cat allergen sensitization are scarce and heterogeneous, with estimates deriving from population and cohort studies. Current estimates range from 5% to 30% of the population and present significant variation across countries [10, 26, 27]. In the United States, it is estimated that 15% of adults and children are sensitized to cats [28]. In Asia, sensitization rates to cats can vary from 8% to 23% across regions [29, 30]. In Europe, the rate of sensitization to cats is higher in certain Northern European countries, such as Denmark and Finland (up to 49%), and comparatively lower in Central and Mediterranean countries, including Belgium, Austria, Italy, or Spain (12%–21%) [10, 31, 32, 33]. Globally, prevalence has increased in recent decades, driven by an increasing number of cat owners in industrialized countries [34, 35].

Numerous studies have investigated the potential correlation between childhood exposure to pets and the risk of developing allergic diseases later in life. However, although most studies appear to support the view that early exposure to cats and other furry animals may exert a protective effect in adolescence and adulthood, inconsistent results have been reported, and no consensus has been reached so far [16]. Some early studies seemed to indicate that pet ownership in early life does not increase or reduce the risk of asthma or AR symptoms in children aged 6–10 [36], but other studies showed that dog or cat ownership during the first years of life reduces the risk of sensitization to dog or cat allergens later in life [37, 38, 39, 40, 41, 42]. In agreement with this view, a recent review of the literature suggested a protective effect against allergy and asthma related to the number of pets owned early in childhood [43]. In contrast, a large meta‐analysis of more than 77,000 children from the EU Child Cohort Network showed that, although early‐life cat and dog ownership did not increase the risk of school‐age asthma, pet exposure could potentially exacerbate risks associated with cat‐ and dog‐specific allergic sensitization [44].

## 3 Cat Allergy Diagnosis

Diagnosing cat allergy typically involves a careful evaluation of the patient's medical history regarding allergies, followed by a physical examination and diagnostic tests. A detailed analysis of the patient's symptoms, including their frequency, duration, and exposure to cats, helps identify any correlation between the presence of cats and allergic reactions. In most cases, a positive skin prick test (SPT) indicates the presence of cat‐specific IgE antibodies. Specific IgE (sIgE) blood tests should be conducted in all patients to confirm the diagnosis, and particularly in patients who cannot undergo SPT due to severe skin conditions or who are at high risk of anaphylaxis, in those who use medications that interfere with skin testing, and in those with a high suspicion of respiratory allergic diseases and a negative SPT.

Advanced diagnostic methods, such as component‐resolved diagnostics (CRD), have enhanced the accuracy of allergy testing [45, 46]. CRD enables the identification of specific cat allergens that trigger allergic reactions, making this information particularly relevant in polysensitized patients [10]. It is also essential to differentiate between primary sensitization and cross‐reactivity. Additionally, CRD enables the development of a personalized treatment plan [27]. It should be noted that CRD and SPT results can differ, as SPT may not correctly identify all sensitized patients, depending on the potency and composition of the SPT allergen extract. Thus, in a recent study, 16.1% of patients positive for cat allergens by CRD were SPT‐negative [47]. Also, multiple studies have directly compared CRD for cat allergy with whole‐extract sIgE testing, showing substantial complementarity but also important differences [48, 49, 50]. As with SPT, whole‐extract sIgE can be positive when tested components are negative (suggesting other minor/unmeasured components, or extract variability), while CRD sometimes identifies sensitization in patients with low or negative extract sIgE [48].

## 4 Cat Allergens

To date, eight cat allergens have been recognized by the World Health Organization/International Union of Immunological Societies (WHO/IUIS) Allergen Nomenclature Sub‐Committee [51] (Table 1). The major allergen is Fel d 1, a small heterodimeric uteroglobin protein produced in the cat's sebaceous, salivary, lachrymal, and anal glands, which spreads to skin and fur during grooming [52, 55]. It is estimated that Fel d 1 is responsible for IgE‐mediated sensitizations in over 90% of cat‐allergic patients and accounts for 60%–90% of the total anti‐cat IgE [52, 53, 54, 55]. The production of Fel d 1 is influenced by cat breed and testosterone levels, with neutered male cats producing less quantity [20]. In addition to Fel d 1, other allergens include Fel d 2 to Fel d 8, with Fel d 4 (a lipocalin found in cat saliva) also playing a significant role in allergy. Fel d 3 (a cystatin A), Fel d 4, and Fel d 7 (a lipocalin) show IgE reactivity in over 50% of cat allergic patients, indicating the importance of detecting additional allergens in cat allergy [53, 56]. A study in Europe showed that Fel d 4 and Fel d 7 were recognized by more than 65% of patients with respiratory allergy, and Fel d 2 (an albumin) was recognized by 30% [54]. Fel d 4, 7, and 8 (a latherin‐like protein) have been identified as the main contributors to the non‐Fel d 1 IgE binding response and elicited inflammatory Th2 cytokines to a similar degree as Fel d 1 [57]. In another study, levels of IgE to Fel d 4 and Fel d 2, but not to Fel d 1 or cat extract, were independently associated with type‐2 biomarkers and total IgE in young people with asthma [58]. Fel d 6 IgE have been detected in 38% of patients with cat allergy, but without clinical relevance [27].

**TABLE 1:** Cat allergens.

Allergen	Protein (molecular mass)	Sensitization prevalence	Clinical significance
Fel d 1	Uteroglobin, chain 1 (38 kDa)	90%–95%	Major cat allergen. Strongly associated with asthma, rhinitis, and conjunctivitis. Primary target of most AIT studies.
Fel d 2	Serum albumin (69 kDa)	20%–30%	Minor allergen. Important for cross‐reactivity with other mammalian albumins (dog, horse, cow).
Fel d 3	Cystatin‐A (11 kDa)	10%–50%	Minor allergen.
Fel d 4	Lipocalin (22 kDa)	< 65%	Clinically associated with asthma and rhinitis, sometimes independent of Fel d 1. Plays a role in cross‐mammal allergy, often correlating with dog Can f 6, and horse Equ c 1.
Fel d 5	IgA (400 kDa)	Low	Low clinical relevance.
Fel d 6	IgM (800–1000 kDa)	< 40%	Low clinical relevance but limited data; rare sensitizer.
Fel d 7	Lipocalin (17.5 kDa)	40%–50%	Clinically important, after Fel d 1 and Fel d 4.
Fel d 8	Latherin‐like protein (24 kDa)	Low	Low clinical relevance but could be important in polysensitized patients.

*Source:* Refs. [27, 51, 52, 53, 54].

Sensitization to Fel d 4 has been found to be associated with asthma in several studies [10, 59, 60, 61]. In a Swedish cohort of 16–25‐year‐olds, Fel d 4 sensitization carried an 11‐fold increased risk of asthma, more than twice the risk associated with Fel d 1, and was also associated with higher rates of combined asthma‐rhinitis and uncontrolled asthma than Fel d 1 sensitized individuals [59]. Parallel Swedish studies in general populations of adolescents and young adults (aged 19 years) similarly linked Fel d 4 to rhinoconjunctivitis, wheezing, and asthma [60, 61].

The patient's cat allergen sensitization profile is highly variable and can be associated with characteristics and severity of the allergic disease [62]. For example, higher concentrations of IgE to Fel d 2 and Fel d 4, but not Fel d 1, could be found in children with cat allergy and atopic dermatitis, suggesting that differences in sensitization to cat allergens in these children could help identify those at increased risk for disease progression and development of asthma [63]. Also, co‐sensitization to Fel d 1 and Fel d 4, but not Fel d 2 was found strongly associated with asthma and asthma symptoms like wheeze [63, 64]. These studies suggest that the implementation of CRD in cat allergy may help clinicians evaluate clinical symptoms and predict their severity [59, 65]. For example, sensitization to Fel d 1 early in childhood can be predictive of having asthma and rhinoconjunctivitis symptoms in adolescence [27, 66]. CRD could also potentially aid in the detection of atypical sensitization profiles and help develop strategies for therapeutic interventions [49]. Currently, CRD testing include Fel d 1, Fel d 2, Fel d 4, and Fel d 7 [67].

In addition to sensitization to cat allergens, numerous recent studies have highlighted the high frequency of polysensitization among individuals with cat allergy, most commonly to dogs, house dust mites, and grass pollen allergens. In a study in China, 46.5% of patients were sensitized to at least two different animal allergens simultaneously (cat, dog, horse) [68], and polysensitization reached 61.1% and 75.9% in studies conducted in Lithuania and Spain, respectively [47, 69]. The cat allergen Fel d 4 cross‐reacts with the horse allergen Equ c 1 and the dog allergen Can f 6, which is also recognized by the IgE of more than 50% of cat‐allergic patients [56]. Cat (Fel d 2), dog (Can f 3), and horse (Equ c 3) serum albumins are highly cross‐reactive, and the galactose‐alpha‐1,3‐galactose (alpha‐gal) present on Fel d 5 (an immunoglobulin A) may be responsible for meat food allergy (pork‐cat syndrome and alpha‐gal syndrome, respectively) [10, 67, 70]. Furthermore, polysensitization to dog and cat allergen components has been linked to the presence of asthma and an increased severity of rhinitis symptoms [50, 71].

In summary, although Fel d 1 is the primary cat allergen, other cat antigens can be determinants, making component‐resolved diagnosis an essential tool in characterizing patients.

## 5 Cat Allergy Management

As with other allergic diseases, allergen avoidance is a first‐line objective for patients with cat allergy. Avoiding direct contact with cats can be the most effective way to prevent or reduce symptoms. Still, it is not always possible, due to the ubiquity of allergens and the fact that pet relinquishment can be traumatic [35]. Most patients do not follow the advice to remove the cat from their home. High‐efficiency particulate air (HEPA) air cleaners can reduce airborne levels of cat allergens in homes with cats. Allergen destruction on filtration media enhances air purifier efficacy but clinical improvement has not been clearly demonstrated [19, 72, 73, 74, 75]. Also, this approach is limited, as allergens are highly volatile, ubiquitous, and persistent even after months of removing the cat from the home [19, 20]. In most cases, allergen avoidance is not a realistic solution for people with cat allergy, whether they are owners or not. Other approaches consisted of treating cats themselves to reduce their allergenicity, the use of diets, and the creation of hypoallergenic breeds [76]. Preliminary studies showed that immunization of cats using recombinant Fel d 1 combined with cucumber mosaic virus‐like particles as a vaccine induced a sustained response with neutralizing Fel d 1–specific IgG and led to lasting relief of symptoms in cat owners with increased tolerance to contact with their animals [77, 78]. Alternatively, the effectiveness of a diet supplemented in anti‐Fel d 1 IgY extracted from egg yolk has shown promising results in reducing Fel d 1 levels in cats [79], although further clinical studies are needed to confirm these findings. Finally, some cat breeds, such as Siberians or Balinese, are believed to produce lower levels of Fel d 1, though all cats studied thus far produce this allergen [20]. Recently, the creation of Fel d 1 chain 2 genome‐edited cats using the CRISPR‐Cas9 system was reported, opening the door to the creation of hypoallergenic breeds [80].

Pharmacotherapy for treating patients allergic to cats has traditionally been based on symptom‐relieving medications [81]. The standard pharmacologic options available to target inflammation symptoms are antihistamines, topical and systemic corticosteroids, mast cell stabilizers, leukotriene antagonists, anticholinergics, and β‐adrenergic agonists.

Allergen immunotherapy (AIT) is another therapeutic option that has demonstrated substantial benefits for treating IgE‐mediated allergies driven by pollen and house dust mites [82]. AIT has been used for the treatment of patients with cat allergy for more than 40 years [83], and, as a disease‐modifying therapy, it has the potential to reduce the need for long‐term medication administration and to prevent the development or progression of asthma. However, prescriptions for AIT to animal dander account for only 3%–5% of all AIT prescriptions; of these, cat AIT accounts for almost 80% of AIT prescriptions involving animal allergens [84].

## 6 AIT and Other Approaches for Treating Cat Allergy

Numerous studies of AIT (mainly subcutaneous [SCIT]) based on standardized cat allergen extracts have demonstrated improvement in symptoms and immunologic changes [85, 86, 87, 88, 89, 90, 91, 92, 93, 94, 95, 96, 97, 98] (Table 2). A real‐life study of patients with rhinitis and/or asthma to cat (*n* = 46) and dog dander (*n* = 20) treated with SCIT revealed significant improvements after 6 and 12 months in respiratory capacity (FEV1), symptoms of rhinitis and asthma, quality of life, the use of medication, and asthma control [96]. These results were observed even when the patient maintained contact with the cat at home. In this study, initial up‐dosing consisted of 3 infusions using a pump at weekly intervals (rush protocol), followed by monthly subcutaneous injections of allergen extracts. In a second study including 61 patients (40 allergic to cat and 21 to dog), an ultra‐rush up‐dosing phase with a 4‐h infusion was tested, followed by monthly subcutaneous injections of allergen extracts, and showed similar clinical benefits at 1, 3, and 6 months [97]. In a pilot study, SCIT demonstrated a positive impact on the quality of life and self‐reported satisfaction in patients with cat allergy [98]. In contrast, studies using cat sublingual AIT (SLIT) are scarce [99, 100]. A double‐blind, randomized controlled trial (RCT) assessed the efficacy of cat SLIT on 50 patients with cat rhinoconjunctivitis with or without asthma for one year, showing a marked reduction (62%) in symptoms in patients receiving SLIT compared to those with placebo [100].

**TABLE 2:** Clinical studies with cat allergen extracts for AIT (1982–2025).

Reference	Study type and population	Treatment	Cat extract composition in active groups	Main results with active treatment
SCIT				
Ohman et al. (1984) [86]	RDBPC 17 pts with AA to cat (9 SCIT, 8 PCB; mean age 28 yo)	SCIT, 4 months	Cat albumin (300 μg/mL)	Significant reduction in bronchial sensitivity (*p* < 0.05) and prick test titer (*p* < 0.01) In addition, significant delay in the onset of ocular (*p* < 0.05) and pulmonary (*p* < 0.02) symptoms on exposure to living cats
Sundin et al. (1986) [87]	RDBPC 39 pts with AA to cat and/or dog (22 SCIT, 17 PCB; 8 to 47 yo)	SCIT, 1–3 years	Fel d 1 (4.3 μg/mL), cat albumin (46.8 μg/mL) (ALK‐Abelló, Denmark)	Significant increase in bronchial tolerance to cat allergen, sustained after 3 years Steady decrease in skin reactivity to allergen extracts during treatment Increase then decrease in allergen‐specific IgE levels and significant increase in IgG levels (no change in PCB pts)
Hedlin et al. (1986) [88]				
Hedlin et al. (1991) [90]				
Van Metre et al. (1988) [89]	RDBPC 22 pts with AA to cat (11 SCIT, 11 PCB; 21 to 52 yo)	SCIT, 1 year	Fel d 1 (21 μg), cat albumin (9 μg/mL) (ALK‐Abelló, Denmark)	Significant decrease in skin and bronchial responses to cat extract Significant increase in IgE and IgG antibodies to cat extract, Fel d 1, and cat albumin, in comparison to PCB
Haugaard and Dahl (1992) [91]	RDBPC 24 pts with AA to cat and/or dog (15 SCIT, 9 PCB; mean age 28 yo) for 5 months, then 19 pts on SCIT for 12 months	SCIT, 12–18 months	Fel d 1 (200 μg/mL) (ALK‐Abelló, Denmark)	Significant decrease in sensitivity to cat during bronchial allergen challenge after 5 months and 1 year (*p* = 0.04 and *p* = 0.003, respectively) Significant decrease in bronchial sensitivity to histamine after 1 year (*p* = 0.02)
Álvarez‐Cuesta et al. (1994) [92]	RDBPC 28 pts with ARC with AA to cat (14 SCIT, 14 PCB; 15 to 65 yo) completed study	SCIT, 1 year	Fel d 1 (maintenance dose:13.2 μg) (Abelló, Spain)	Significant improvement in medication‐symptom score compared to PCB (*p* < 0.001) Significant improvement in SPT (*p* < 0.001), conjunctival provocation test (*p* < 0.001), and allergen bronchial provocation test (*p* < 0.05)
Varney et al. (1997) [93]	RDBPC 28 pts with ARC with or without AA to cat (13 SCIT, 15 PCB; ≥ 18 yo)	SCIT, 3 months	Fel d 1 (15 μg/mL; maintenance dose: 15 μg) (Alutard SQ, ALK‐Abelló, Denmark)	Significant reduction in symptoms during cat exposure (*p* < 0.001), with no change in the PCB group Reduction in peak flow response to cat exposure (*p* < 0.005) as well as reductions in conjunctival provocation sensitivity, and skin sensitivity to cat extract
Ewbank et al. (2003) [94]	RDBPC 28 pts with AR with or without AA to cat (21 SCIT, 7 PCB; ≥ 18 yo) completed study	SCIT, 1 year	Fel d 1 (0.6 μg, 3.0 μg, or 15.0 μg at maintenance) (ALK‐Abelló, CT, USA)	Dose‐dependent differences in SPT, cat‐specific IgG4 and reduction in CD4+/IL‐4+ PBMCs, versus PCB Significant decrease in SPT sensitivity with 3.0 and 15.0 μg Fel d 1 (*p* = 0.02 and *p* = 0.002, respectively), and significant increase in cat‐specific IgG4 (*p* = 0.01 and *p* = 0.006, respectively) Significant reduction in the percent of CD4+/IL‐4+ PBMCs only with 15.0 μg Fel d 1 (*p* = 0.003)
Nanda et al. (2004) [95]	RDBPC 28 pts with AR with or without AA to cat (≥ 18 yo) 26 pts completed study (20 SCIT, 6 PCB)	SCIT, 1 year	Fel d 1 (0.6 μg, 3.0 μg, or 15.0 μg at maintenance)	Dose‐dependent differences in total symptom scores on nasal challenge (*p* < 0.0001), titrated SPT (*p* < 0.0001), and cat‐specific IgG4 (*p* = 0.003), versus PCB
Uriarte and Sastre (2020, 2022) [96, 97]	Observational, prospective 46 pts with AR or AA to cat (mean age 34 yo)	SCIT, 1 year	Fel d 1 (15 μg/mL; maintenance dose: 15 μg) (Alutard SQ, ALK‐Abelló, Denmark)	Significant improvement in FEV1, in AR and AA symptoms, VAS, ACT, medication, and QoL (ESPRINT‐15, AQLQ) scores observed at 6 months and continued at 12 months 8.1% of SCIT doses triggered a systemic reaction, 5.4% produced a local reaction
Colque‐Bayona et al. (2025) [98]	Prospective, single‐arm, longitudinal pilot study 13 pts with ARC with or without AA to cat and/or dog (mean age 32 yo)	SCIT, 1 year	Various commercial extracts	Significant improvement in the NRS for ocular and nasal symptoms (*p* = 0.001 and *p* = 0.002, respectively), and in mean ACT score (*p* = 0.011) Marked decrease in antihistamine and SABA use Improvement in QoL for rhinitis according to the ESPRINT‐15 questionnaire (*p* = 0.003); improvement in QoL for asthma in 70% patients, although no significant change in AQLQ
SLIT				
Nelson et al. (1993) [99]	RDBPC, in a cat EEC 41 pts with ARC with or without AA to cat (20 SLIT, 21 PCB; 18 to 74 yo)	SLIT, 105 days	Cat hair and epithelium extract 100,000 AU/mL (Fel d 1: 10–20 units/mL, Hollister‐Stier; maintenance dose 450 to 900 Fel d 1 units)	No differences were observed between the active and PCB groups
Álvarez‐Cuesta et al. (2007) [100]	RDBPC, in a cat EEC 50 pts with ARC with or without AA to cat (25 SLIT, 25 PCB; mean age 27 yo) 33 pts completed the study	SLIT, 1 year	Fel d 1 (0.51 μg/mL; maintenance dose: 17.1 μg) (LETI, Spain)	Significant reduction (62%) in symptoms during the NCT (*p* < 0.001), while no changes in the PCB group Significant reduction in PEF response to cat exposure (*p* < 0.05), and significant improvement in SPT reactivity (*p* < 0.05), without significant changes in the PCB group

Abbreviations: AA, allergic asthma; ACT, asthma control test; AIT, allergen immunotherapy; AQLQ, asthma quality of life questionnaire; AR, allergic rhinitis; ARC, allergic rhinoconjunctivitis; AU, allergy unit; EEC, environmental exposure chamber; FEV1, forced expiration volume in 1 s; NCT, natural exposure challenge test; NRS, numerical rating scale; PBMCs, peripheral blood mononuclear cells; PCB, placebo; PEF, peak expiratory flow; pts, patients; QoL, quality of life; RDBPC, randomized double‐blind placebo‐controlled; SABA, short‐acting ß2‐agonist; SCIT, subcutaneous immunotherapy; SLIT, sublingual immunotherapy; SPT, skin prick test; VAS, visual analogue scale; yo, years old.

As the use of cat dander extracts in SCIT and SLIT has generally presented difficulties with standardization and side effects, two different Fel d 1‐targeting approaches have been investigated, one based on peptides and the other on monoclonal antibodies (mAbs) (Table 3). The peptide immunotherapy approach involved intradermal injection of Cat‐PAD (Cat Peptide Allergy Desensitization), an equimolar mixture of seven MHC class II‐restricted, T cell‐targeted peptides of 13–17 amino acids covering the C‐terminal sequence of chain 1 from Fel d 1 [101]. Cat‐PAD was tested in a double‐blind dose‐finding RCT [102], which demonstrated an improvement in the ocular and nasal components of rhinoconjunctivitis symptoms of cat allergy persisting 1 year after the start of treatment, and even after 2 years [103]. More recently, the mAb approach has consisted of passive subcutaneous administration of REGN1908 and REGN1909, two fully human IgG4 mAbs that bind to different regions of Fel d 1, thereby blocking IgE binding and inhibiting basophil activation in cat‐allergic individuals [104, 105, 106, 107]. However, despite the initial promise, the success of approaches targeting only Fel d 1 for the control of allergy symptoms remains uncertain [76].

**TABLE 3:** Clinical studies using Fel d 1‐targeting approaches.

Reference	Study type and population	Treatment	Main results
Worm et al. (2011) [101]	RDBPC 88 pts with ARC with or without mild AA to cat (66 Active, 22 PCB; 18 to 65 yo)	Fel d 1 synthetic peptides (Cat‐PAD, 0.03 to 12 nmol ID or 1 to 20 nmol SC) (Circassia, UK)	The peptide vaccine comprising the immunodominant regions of the allergen was safe and well tolerated when given to subjects with cat allergy as a single dose The dose of vaccine resulting in the greatest reduction in late‐phase skin response was defined as 3 nmol for future clinical development
Patel et al. (2013) [102]	RDBPC, in cat EEC 1‐year follow‐up 89 pts with ARC with or without mild AA to cat (53 Active, 36 PCB; 18 to 65 yo)	Fel d 1 synthetic peptides (Cat‐PAD, 8 × 3 nmol or 4 × 6 nmol ID), 3 months (Circassia, UK)	Significantly greater improvement in ARC symptom scores with 4 × 6 nmol dose persisting 1 year after the start of treatment versus 8 × 3 nmol dose (*p* = 0.0342) and PCB (*p* = 0.0104)
Couroux et al. (2015) [103]	RDBPC, in cat EEC 2‐year follow‐up 51 patients with ARC with or without mild AA to cat (39 Active, 12 PCB; 18 to 65 yo)	Fel d 1 synthetic peptides (Cat‐PAD, 8 × 3 nmol or 4 × 6 nmol ID), 3 months (Circassia, UK)	2 years after the start of treatment, the 4 × 6 nmol dose showed a non‐significant reduction in total ARC symptom scores compared to PCB (*p* = 0.13) on days 2–4 after EEC challenge (primary endpoint), but this difference was significant at the end of day 4 (secondary endpoint) when the cumulative allergen challenge was greatest (*p* = s0.02) No meaningful effect of 8 × 3 nmol dose
Orengo et al. (2018) [104]	RDBPC 73 pts with AR to cat (36 Active, 37 PCB; mean age 28 yo)	Anti‐Fel d 1 mAbs (REGN1908/1909 600 mg SC), 85 days	A single dose of blocking IgG reduces clinical symptoms in response to nasal provocation (*p* = 0.0003)
Shamji et al. (2021) [105]	RDBPC 73 pts with AR to cat (36 Active, 37 PCB; mean age 28 yo)	Anti‐Fel d 1 mAbs (REGN1908/1909 600 mg SC), 85 days	Type 2 cytokines (IL‐4, IL‐5, and IL‐13) and chemokines (CCL17/TARC, CCL5/RANTES [regulated upon activation, normal T‐cell expressed and secreted]) in nasal fluid were inhibited in the Active group compared with PCB (*p* < 0.05 for all) IL‐13 and IL‐5 concentrations correlated with total nasal symptom score improvement
de Blay et al. (2022) [106]	RDBPC, in cat EEC 56 pts with AR/C and mild AA to cat (29 Active, 27 PCB; mean age 29 yo)	Anti‐Fel d 1 mAbs (REGN1908/1909 600 mg SC), 85 days	Single‐dose REGN1908/1909 significantly prevented reductions in FEV1 from day 8 to day 85, compared to PCB Active group pts did not have any early asthmatic responses by 4 hours; PCB pts had early asthmatic responses within 1 h Active group pts tolerated 3‐fold higher allergen quantities (*p* < 0.05 at all time points) versus PCB Active group pts reduced skin test reactivity to cat allergen versus PCB at all time points tested (nominal *p* < 0.001)

Abbreviations: AA, allergic asthma; AR, allergic rhinitis; AR/C, allergic rhinitis with/without conjunctivitis; EEC, environmental exposure chamber; FEV1, forced expiration volume; ID, intradermally; mAbs, monoclonal antibodies; PCB, placebo; pts, patients; RDBPC, randomized double‐blind placebo‐controlled; SC, subcutaneously.

Alternatively, Fel d 1 allergen presentation on virus‐like particles (VLPs) tested in mice and human basophils in vitro resulted in increased immunogenicity with reduced reactogenicity, suggesting such a design might be safe and effective [108]. Another study showed that Fel d 1 displayed on VLPs strongly induced a protective IgG response compared to free allergen, while at the same time failed to induce mast cell activation [109]. Recently, a platform based on plant‐made bioparticles displaying recombinant Fel d 1 was tested in vitro, demonstrating that the allergen‐covered particles were potent immune activators of human monocyte‐derived dendritic cells, and also that their allergenicity was lower than aluminum‐adsorbed natural Fel d 1 allergens [110]. These technologies could be a promising AIT approach to both enhance immunogenicity and reduce the risk of anaphylaxis.

Combination treatments of AIT with monoclonal antibodies could be a promising avenue for future research, as they promise better tolerance, increased safety, and overall improved efficacy, as observed in food or house dust mite‐derived allergies [111, 112, 113]. In a randomized controlled trial, the monoclonal antibody tezepelumab (anti‐thymic stromal lymphopoietin, TSLP) was used in combination with SCIT in patients with allergic rhinitis due to cat allergy [114]. The results showed that inhibition of TSLP, which plays a central role in the initiation and persistence of allergic responses, augmented the efficacy of SCIT during therapy and promoted tolerance after a 1‐year course of treatment.

All in all, however, the above results suggest that a single allergen, such as Fel d 1, might not be sufficient to treat cat allergy, at least with the platforms used in these studies. Therefore, a broader approach involving more than one allergen could be required for the effective treatment of cat allergy. In subjects with a positive SPT to cat extract, patterns of sensitization to different cat allergens are variable and can be complex, depending on whether the subjects are also sensitized to dog and/or horse, as well as cat ownership [46, 115]. In the case of cat AIT, a nasal challenge test could help refine the pet allergy profile and document clinically meaningful sensitization [17, 49]. Additionally, the identification of IgE anti‐Fel d 1 as the primary sensitizing allergen is critical [116]. However, as described previously, the presence not only of Fel d 1 but also of other cat allergens, especially Fel d 4, is expected to play a significant role in the observed efficacy of AIT [100]. Incidentally, the potential importance of non‐Fel d 1 allergens may explain, at least in part, why some of the aforementioned approaches focusing solely on Fel d 1 ultimately proved unsuccessful. Including a broad spectrum of cat allergens in AIT products is therefore expected to enhance their efficacy, as it caters to the diverse allergen sensitivities among patients. In this regard, a recent study evaluated two cat SLIT liquid formulations (Staloral Cat 300 IR/mL, Stallergenes Greer, France; and Osiris Cat 300 IR/mL, ALK‐Abelló, Denmark), to quantify Fel d 1 and Fel d 4 allergens and to identify other allergens such as Fel d 2, Fel d 3, and Fel d 7 (Fel d 8 could not be detected) [117]. This study found that both cat allergen extracts were qualitatively similar, presenting similar protein and allergen profiles. However, despite being labeled with a same unit (Index of Reactivity or IR) but based on a different definition, the extracts differed significantly in total allergenic activity (TAA), with Staloral Cat 300 IR/mL displaying 2 times higher TAA than Osiris Cat 300 IR/mL (according to an in‐house method) and contained 1.6 times more proteins (1.3 and 1.9 times more Fel d 1 and Fel d 4, respectively) [117]. Although no conclusions on clinical efficacy or safety can be drawn from these data, this study suggests that allergen composition and potency should be taken into consideration when prescribing AIT extracts. Such a high‐potency product would overcome the effects of non‐Fel d 1 allergens in the disease, including Fel d 4, which is especially relevant in patients with allergic asthma [10].

One of the primary objectives of using AIT in patients with AR is to prevent disease progression to asthma or to prevent asthma exacerbations [82]. Recently, the results of a real‐world evidence study (EfficAPSI) with the largest number of person‐years of follow‐up to date in the field of AIT were published [118]. This study evaluated the impact of STG IR‐SLIT‐liquid (Staloral with diverse allergen extracts, Stallergenes Greer) on asthma onset and evolution in 112,492 patients with AR (vs. 333,082 non‐AIT controls) of whom 1902 patients were dispensed STG Cat IR‐SLIT‐liquid. The results showed that STG Cat IR‐SLIT‐liquid was significantly associated with a reduction in the risk of asthma onset or worsening in cat allergic patients (adjusted HR = 0.80; 95% CI, 0.76–0.85 using a sensitive definition of asthma events, which considered the first dispensation of an asthma medication or a hospitalization for asthma or a long‐term disease for severe asthma). Compared to controls, exposure to STG Cat IR‐SLIT‐liquid reduced asthma onset (adjusted HR = 0.82; 95% CI, 0.76–0.89) in patients with cat AR without asthma at baseline (*n* = 969). In those with pre‐existing asthma at baseline (*n* = 933), STG Cat IR‐SLIT‐liquid prevented asthma worsening compared to controls (adjusted HR = 0.64: 95% CI, 0.59–0.69) [118]. In conclusion, the large real‐world study yielded promising results for the use of AIT in patients with cat allergy, regardless of whether they had pre‐existing asthma. Furthermore, as the Cat SLIT‐liquid product dispensed in this study contained 100 IR/mL of cat allergen extract, this suggests a positive trend that could be improved with the 300 IR/mL formulation first launched in France in November 2021 [117]. STG 300 IR Cat SLIT‐liquid appears to be well‐tolerated [119]. As of November 2024, this product presented a cumulative rate of safety reports (as a function of the total number of patients exposed) of 1.37% (265/19,422). Most adverse drug reactions were application‐site reactions, such as mouth edema or throat irritation. Severe adverse reactions occurred in 5.3% of cases, but there were no reports of anaphylaxis, severe local allergic reactions, or eosinophilic esophagitis.

## 7 Cat AIT—Challenges and Current Developments

When faced with cat allergy, the three options that are theoretically available are avoidance of cats and their allergens, pharmacological treatment of symptoms, and AIT. Given the ubiquitous presence of cats in households and the emotional bonds between cat owners and their pets, allergen avoidance, although often ineffective in many cases, is often impractical or impossible [35]. The context of exposure to cats, the patient's environment, and personal expectations regarding treatment all play a key role in the management approach adopted by physicians, making a case‐by‐case approach necessary. The high risk of asthma onset should also be considered when managing patients with cat allergy [120]. In personalizing treatments, physicians must pay special attention to the allergy profile of each patient using component‐resolved diagnostics and consider poly‐sensitization to other aeroallergens. In this way, healthcare providers can maximize effectiveness while minimizing side effects, ensuring the best quality of life for the patients.

The high prevalence of allergic diseases and cat allergy globally makes the development of more efficient and safe AIT even more essential [10, 116, 121, 122]. This is especially necessary for patients with asthma, as their disease can often be uncontrolled by standard drugs [120]. Although numerous innovative solutions have been proposed in recent years, some challenges and barriers still limit the widespread use of AIT for cat allergy (Table 4). As previously discussed, comparing the numerous cat dander extracts available for AIT on the market is challenging due to a lack of proper standardization. In vitro evaluation using allergen immunoassays is important because cat dander extracts may vary in the quality and quantity of certain individual allergen components and other molecules [67]. Additionally, given the differences in units used, the variations between extracts could impact allergenic potency and ultimately affect AIT success [117].

**TABLE 4:** Summary of challenges and solutions to effective treatment of cat allergy with AIT.

Challenges and barriers	Solutions
*Allergen extract standardization and potency*. Variability in source material and manufacturing leads to inconsistent potencies and dosing, making it challenging to ensure that each patient receives an optimal, reproducible dose of the cat allergen [117, 123]. *Molecular complexity of extracts*. Although Fel d 1 is the dominant cat allergen, other allergens (notably Fel d 2 and Fel d 4) can be critical to allergic disease. *Safety concerns and risk of adverse reactions*. Cat SCIT has been associated with a risk of systemic reactions. SLIT is generally safer, but local oral side effects could affect long‐term adherence. *Patient adherence and persistence*. AIT requires a long‐term commitment (3 years) for optimal results. In the case of SLIT, the treatment is daily. Common reasons for poor adherence include injection frequency (SCIT), treatment duration, travel/logistical burdens, perceived slow onset of benefit, and cost [124, 125]. *Accessibility and cost*. Specialist AIT services are concentrated in urban centers, and the production of standardized cat‐allergen extracts is costly; insurance coverage may be limited [126]. *Persistent environmental exposure*. Fel d 1 exposure is ubiquitous and complex to remove effectively. Environmental control (e.g., HEPA filtration) can be expensive and often only partially successful. AIT efficacy could be reduced when background allergen levels remain high. *Limited and heterogeneous clinical evidence for cat‐specific AIT*. Unlike pollen, cat dander is present year‐round. Trials must therefore incorporate a pre‐treatment baseline period to quantify ongoing symptom and medication use, lengthening study duration and increasing variability in outcome measures [127]. *Lack of objective biomarkers to guide and monitor therapy*. Patient‐reported symptom scores and rescue medication use drive treatment decisions and AIT duration. There are no broadly validated in vitro biomarkers (e.g., functional IgG4 assays, cytokine profiles) that reliably predict which patients will respond best or when therapy can be safely stopped [123, 128].	*Improved extract standardization*. Detailed characterization and standardization of extracts would enhance the credibility of AIT among physicians and help understand effects in patients [117, 129]. *Component‐resolved diagnostics*. CRD is a highly relevant tool that should be used to properly characterize the immunologic profile of patients with cat allergy [27]. *Enhanced patient‐support programs to improve adherence*. Combining patient‐, provider‐, and society‐based actions could be helpful to enhance adherence to AIT, but studies on this are limited [130, 131]. Also, digital technologies such as integrated smartphone apps with electronic diaries allowing real‐time symptom tracking and feedback can improve adherence [132]. Another potential solution is to connect “smart” dispensers to provide reminders and a record of SLIT administration [133]. *Enhanced patient education*. Patients need to understand the importance of adequate and sustained treatment for their allergy. Patient education by allergists or other healthcare providers should be a priority [134]. Shared decision‐making can bolster adherence to the treatment. Patient support forums where patients share experiences, tips for managing local reactions, and encouragement can reduce feelings of isolation and promote long‐term commitment. Cost‐effectiveness studies should support coverage. *Rigorous clinical trials focused on cat allergy populations*. Compared with other allergies, there are a limited number of large trials focused on the population with cat allergy. *Development of predictive biomarkers for personalized AIT*. By pre‐selecting patients whose biomarker profiles (e.g. high–affinity Fel d 1–specific IgE, basophil activation thresholds) correlate with a higher likelihood of response, costs, duration, and exposure of AIT non‐responders could be reduced [128]. Predictive biomarkers can personalize patient care, improve safety, and support financing of therapies [135].

Abbreviations: AIT, allergen immunotherapy; CRD, component‐resolved diagnostic; HEPA, high‐efficiency particulate air; SCIT, subcutaneous immunotherapy; SLIT, sublingual immunotherapy.

Implementation of AIT for the treatment of cat allergy has been hindered by a lack of data from extensive and conclusive clinical trials [121]. One of the reasons for this situation is that AIT trials must incorporate a pre‐treatment baseline period to quantify ongoing symptoms and medication use, lengthening study duration and increasing variability in outcome measures [127]. Also, demonstrating statistically significant differences often requires large and homogeneous population sizes to achieve adequate power, which is difficult to achieve in the case of cat allergy. A high placebo response has been observed in some trials, as seen in the Cat‐PAD Phase III trial [102, 103]. In the case of cat SLIT, local oral itching/swelling after active doses could potentially reveal treatment assignment in lengthy trials. As mentioned, non‐Fel d 1 cat allergens, such as Fel d 2 or Fel d 4, contribute to symptoms in subsets of patients, which severely complicates the selection of patients and the therapies used. However, it should be considered that in some early cat AIT studies not all cat allergens were available for molecular diagnosis, or participants were not systematically diagnosed with sIgE. Thus, current associations with rhinitis or asthma may be incomplete and more detailed analyses would provide a better insight into the benefits of AIT for specific populations of patients. In contrast to house dust mites or pollen AIT, no approved component‐resolved (recombinant) formulations exist to personalize AIT for cat allergy. Also, although candidate biomarkers have been investigated for cat AIT, none have been validated for routine clinical use. Current evidence points to potential biomarkers such as Fel d 1–specific IgE, IgG4, and the IgG4:IgE ratio, basophil activation markers (CD63 and CD203c upregulation in response to Fel d 1), Treg‐associated markers (IL‐10, FOXP3), and clinical biomarkers that may help predict or monitor response [128].

Recently, a survey of patients allergic to cat dander who have been or were in the process of being treated with STG 300 IR Cat SLIT‐liquid, composed of multiple allergens beyond Fel d 1, was conducted in France. The objective was to explore the profiles of patients (*n* = 197) and their perceptions of treatment effectiveness. Preliminary results revealed promising trends: nearly 9 out of 10 patients reported symptom improvement, with 60% noting a better overall health perception and a significant +2.3‐point gain on a 0–10 scale [136]. These encouraging outcomes—particularly in controlling rhinitis and reducing asthma exacerbations—highlight the potential of this treatment in daily practice. Full results will be detailed in a forthcoming publication.

Unfortunately, cat allergy is often underestimated and untreated, compared with house dust mite or grass pollen allergies. Cat owners can become accustomed to chronic allergy symptoms rather than recognizing a persistent allergic reaction, especially if these are mild, which can lead to long‐term exposure and a worsening of the allergy over time. Currently, cat AIT represents a small percentage of all AIT prescriptions [84], and it is estimated that a significant proportion of patients who could benefit from AIT are not offered the opportunity for this therapy. This situation is potentially worrisome in patients with asthma or with the potential to develop asthma. Some of the barriers for AIT prescription are a lack of conviction among physicians, a perception of limited effectiveness, and issues with patient adherence (Table 4). Thus, according to physicians, the critical factors to consider when prescribing AIT include the patient's inability/non willingness to avoid cats, high patient motivation, and the need of treatment or prevention of severe symptoms such as asthma or persistent rhinoconjunctivitis. Additionally, from a patient's perspective, AIT can be a challenging experience. AIT requires a long‐term commitment to benefit from its potential disease‐modifying effects and the prevention of disease progression (at least 3 years of treatment is recommended) [82], whereas many patients prefer a quick‐relief solution, especially since allergen avoidance can be difficult. Nevertheless, the promising effectiveness of AIT products, such as STG 300 IR Cat SLIT‐liquid, which features a broader allergen composition and high potency, and/or improved tolerance to AIT (e.g., through combination with specific monoclonal antibodies), may increase the number of patients with cat allergy who could access this therapy.

## 8 Conclusions

Cat allergy is a highly prevalent condition that can become severe if it develops into asthma. Cat allergens, particularly Fel d 1, are potent triggers for asthma symptoms and can lead to severe respiratory issues, primarily due to their protein structure and persistence in the environment. Still, cat allergy is often unrecognized and undertreated. Although AIT has long been a cornerstone in managing cat allergies, offering a disease‐modifying approach by gradually desensitizing the immune system to cat allergens, it is still prescribed at low rates. Some of the barriers to AIT adoption include the scarcity of clinical data on cat AIT, issues with standardizing cat allergen extracts, and challenges to long‐term treatment adherence, among others. It is possible that immunotherapy platforms tested in the past with poor results may have failed due to an exclusive focus on the Fel d 1 allergen. Instead, the design of cat AIT products should reflect the patients' exposure to all distinct cat allergens. Today, new data on a product with an adequate combination of cat allergens at high concentration could ensure the necessary allergenic potency for effective and safe AIT. This will enhance physician and patient confidence, enabling broader access to these therapies.

## Author Contributions

**Pascal Demoly:** writing – review and editing. **Myriam Zakariya:** writing – review and editing. **Ignacio Dávila:** writing – review and editing. **Giuseppe Scibilia:** writing – review and editing. **Valeria Ortolani:** writing – review and editing. **Javier Domínguez‐Ortega:** writing – review and editing. **Karl‐Christian Bergmann:** writing – review and editing. **Philippe Gevaert:** writing – review and editing. **Alain Didier:** writing – review and editing.

## Funding

This work was funded by Stallergenes Greer.

## Conflicts of Interest

Pascal Demoly reports no direct financial interests; he has received institutional grants for teaching and research activities from ALK‐Abelló, AstraZeneca, Chiesi, GlaxoSmithKline, Menarini, Puressentiel, Stallergenes Greer, ThermoFisher Scientific, Viatris, and Zambon.

Myriam Zakariya has no conflict of interest to declare.

Ignacio Dávila has received grants for ThermoFisher Diagnostics, ISCIII, Sanofi, and Junta de Castilla y León; and consulting fees and/or payment for lectures including service on speaker's bureaus from Allergy Therapeutics, AstraZeneca, Diater, GlaxoSmithKline, Leti, MSD, Sanofi; and support for attending meetings and/or travel from Sanofi.

Giuseppe Scibilia has no conflict of interest to declare.

Valeria Ortolani has no conflict of interest to declare.

Javier Domínguez‐Ortega reports having received consulting fees and/or payment or honoraria for lectures, presentations, speakers bureaus, manuscript writing or educational events from ALK‐Abelló, AstraZeneca, GlaxoSmithKline, Leti Pharma, Sanofi and Stallergenes Greer, not related to the article submitted.

Karl‐Christian Bergmann reports having received consulting fees from ALK‐Abelló, AstraZeneca, Bencard, Celltrion, Chiesi, GlaxoSmithKline, Novartis, Sanofi, and Stallergenes Greer, not related to the article submitted.

Philippe Gevaert reports grants, consulting fees and/or payment or honoraria for lectures, presentations, speakers bureaus, manuscript writing or educational events from Eli Lilly, GlaxoSmithKline, Sanofi‐Regeneron and Stallergenes Greer, not related to the article submitted; and support for attending meetings and/or travel from Stallergenes Greer.

Alain Didier reports consulting fees from ALK‐Abelló, AstraZeneca, Chiesi, GlaxoSmithKline, Menarini, Sanofi, Stallergenes Greer, and Viatris, not related to the article submitted; and support for attending meetings and/or travel from ALK‐Abelló, AstraZeneca and Sanofi.

## Data Availability

The data that support the findings of this study are available from the corresponding author upon reasonable request.
